# ﻿Taxonomic dissection based on molecular evidence of the *Eriosycecurvispina* complex (Cactaceae): identifying nine endemic species from Central Chile

**DOI:** 10.3897/phytokeys.237.107403

**Published:** 2024-01-22

**Authors:** Helmut E. Walter, Arón Cádiz-Véliz, Beatriz M. Meriño, Heidy M. Villalobos-Barrantes, Pablo C. Guerrero

**Affiliations:** 1 The EXSIS Project: Cactaceae Ex-Situ & In-Situ Conservation, 31860 Emmerthal, Germany; 2 Departamento de Botánica, Facultad de Ciencias Naturales y Oceanográficas, Universidad de Concepción, Casilla 160C, 4030000, Concepción, Chile; 3 Institute of Ecology and Biodiversity (IEB), Concepción, Chile; 4 Millennium Institute Biodiversity of Antarctic and Sub-Antarctic Ecosystems (BASE), Concepción, Chile; 5 Escuela de Química, Universidad de Costa Rica, CP 11501-2060, San José, Costa Rica; 6 Centro de Investigación en Biología Celular y Molecular, Universidad de Costa Rica, CP 11501-2060, San José, Costa Rica

**Keywords:** Cactaceae, Caryophyllales, central Chile, *Eriosycecurvispina*, Horridocactus, new combinations, succulent plants, taxonomy, Cactaceae, Caryophyllales, Chile central, Eriosycecurvispina, Horridocactus, plantas suculentas, taxonomía

## Abstract

Chile’s distinctive flora, geographical isolation, and complex topography collectively contribute to a notable endemic species diversity, particularly within central regions identified as critical areas for biodiversity conservation. The cactus genus *Eriosyce*, as currently circumscribed, encompasses seven sections, with Eriosycesect.Horridocatus presenting a notably complex species group. This study investigates the *E.curvispina* complex, a member of the Notocacteae tribe common in central Chile, by incorporating new populations and examining phylogenetic relationships using four plastid and one nuclear molecular marker. The phylogenetic analysis of sampled individuals identified nine independent lineages, each warranting recognition at the species rank. Despite minimal morphological differences among taxa, morphological characters were utilized to support and stabilize the DNA-based phylogenetic hypothesis. The results highlight the high taxonomic diversity in these cactus lineages and have implications for the classification of the *E.curvispina* complex, including new combinations and proposals of conservation status.

## Abstract (es)

La flora distintiva de Chile, su aislamiento geográfico y topografía compleja contribuyen colectivamente a una notable diversidad de especies endémicas, particularmente dentro de las regiones centrales identificadas como áreas críticas para la conservación de la biodiversidad. El género de cactus *Eriosyce*, tal como está circunscrito actualmente, abarca siete secciones, presentando Eriosycesect.Horridocatus un grupo de especies notablemente complejo. Este estudio investiga el complejo *E.curvispina*, un miembro de la tribu Notocacteae común en Chile central, incorporando nuevas poblaciones y examinando las relaciones filogenéticas utilizando cuatro marcadores moleculares del cloroplasto y uno nuclear. El análisis filogenético de las poblaciones muestreadas identificó nueve linajes independientes, cada uno mereciendo reconocimiento a nivel de especie. A pesar de las mínimas diferencias morfológicas entre los taxones, se utilizaron caracteres morfológicos para apoyar y estabilizar la filogenia basada en ADN. Los resultados resaltan la alta diversidad taxonómica en estos linajes de cactus y tienen implicaciones para la clasificación del complejo *E.curvispina*, incluyendo nuevas combinaciones y propuestas de estado de conservación.

## ﻿Introduction

Chile has a unique flora with a great diversity of endemic species — a condition given by its geographical isolation from the rest of the South American continent (Arroyo et al. 2008; Scherson et al. 2017). Additionally, the complex topography, with two mountain ranges crossing the country from north to south and west to east, joined by transversal mountain ranges in the center of the country, favors the origin of narrow endemisms (Mardones and Scherson 2023). Central Chile has been identified as one of the priority areas of biodiversity worldwide (Myers et al. 2000) and characterized as one of the world’s centers of cactus diversity (Hernández-Hernández et al. 2011). Many species belong to the tribe Notocacteae, one of the oldest and most diverse lineages in South America and are estimated to have diverged between 16–14.8 Ma (Arakaki et al. 2011; Hernández-Hernández et al. 2014). They are a heterogeneous tribe of mostly globose, small to medium-sized, mostly unbranched stems, and colorful diurnal flowers, with a great variety of forms (Guerrero et al. 2019a, b).

This study focuses on *Eriosyce* Phil., a genus of the Notocacteae with a long taxonomic with a high level of uncertainty, due to the long history of taxonomic changes since Rodulfo A. Philippi (Philippi 1872). In 1994, F. Kattermann merged five former genera (*Islaya* Backeb., *Pyrrhocactus* A. Berger, *Horridocactus* Backeb., *Neoporteria* Britton & Rose, and *Thelocephala* Y. Ito) into *Eriosyce*. The broad circumscription of *Eriosycesensu*Kattermann (1994) was supported as monophyletic, only excluding *Rimacactuslaui* (Lüthi) Mottram) (Guerrero et al. 2019b), and then all five former genera were regarded in synonym of a broad circumscribed *Eriosyce*. The genus *Eriosyce* harbors more than 50 species, has a relatively wide geographic distribution (between the latitudes of 13°–36°S and longitudes between 70°W and 66°W, altitudes ranging from sea level up to 2800 m.a.s.l.), and a large morphological heterogeneity (Guerrero et al. 2019b). Its current circumscription (Guerrero et al. 2019b) includes the following sections: I. Eriosycesect.Eriosyce (Phil.) Katt. (distributed in Chile, Argentina & Peru; 5 accepted taxa), II. Eriosycesect.Campanulatae P.C.Guerrero & Helmut Walter (endemic to Chile; 2 accepted taxa), III. Eriosycesect.Pyrrhocactus (A. Berger) Katt. (endemic to Argentina; 4 accepted taxa), IV. Eriosycesect.Horridocatus (Backeb.) Katt. (endemic to Chile; 12 accepted taxa), V. Eriosycesect.Diaguita P.C.Guerrero & Helmut Walter (endemic to Chile; 3 accepted taxa), VI. Eriosycesect.Neoporteria (Britton & Rose) Katt. (endemic to Chile; 15 accepted taxa), and VII. Unnamed section (endemic to Chile; 22 accepted taxa) (Guerrero et al. 2011a, b, 2019b).

In 1938, Backeberg erected the genus *Horridocactus* for a group of plants with 22 species from southern central to northern Chile based on a single character state – the lack of hairs on the pericarpel and hypanthium (in contrast to his genus *Neochilenia*) (Backeberg 1938). Yet, the monophyly of *Horridocactussensu* Backeberg was not supported by molecular data (Guerrero et al. 2019b). Kattermann (1994) merged Backeberg’s genera *Horridocactus* and *Neochilenia* into his EriosycesectionNeoporteria, subsection Horridocactus, which includes 14 species from south-central to northern Chile. Conversely, the findings of Villalobos-Barrantes et al. (2022) supported the monophyly of the EriosycesectionHorridocactus, which is circumscribed to include the following nine species: *E.armata* (F.Ritter) P.C.Guerrero & Helmut Walter, *E.aspillagae* (Söhrens) Katt., *E.cuvispina* (Bertero ex Colla) Katt., *E.duripulpa* (F.Ritter) P.C.Guerrero & Helmut Walter, *E.engleri* (F.Ritter) Katt., *E.garaventae* (F.Ritter) Katt., *E.jussieui* (Monv. ex Salm-Dyck) P.C.Guerrero & Helmut Walter, *E.limariensis* (F.Ritter) Katt. and *E.napina* (F.Ritter) Katt.

The oldest species in EriosycesectionHorridocactus – *Cactuscurvispinus* Bertero ex Colla – was named by Bertero in 1829. However, the type specimen was lost, thus a neotype was designed by Kattermann (1994). During the last 200 years the species was placed in various genera by former authors: Gay (1847): *Echinocactus*; Britton and Rose (1922): *Malacocarpus*; Berger (1929): *Pyrrhocactus*; Kreuzinger (1941): *Hildmannia*; Backeberg (1940): *Horridocactus*; Donald and Rowley (1966): *Neoporteria* and, Kattermann (1994): *Eriosyce*. Various authors merged species and infraspecific taxa mostly erected by F. Ritter into an “*E.curvispina* complex” along several waves of lumping: Donald and Rowley (1966) merged 19 taxa into *Neoporteriacurvispina* (Bertero ex Colla) Donald & Rowley. The “*E.curvispina* complex” *sensu*Hoffmann (1989) comprised 6 taxa (i.e. Neoporteriacurvispinavar.marksiana (F.Ritter) A. Hoffmann, N.curvispinavar.lissocarpa (F.Ritter) Donald & Rowley, N.curvispinavar.engleri (F.Ritter) A. Hoffmann, N.curvispinavar.andicola (F.Ritter) Donald & Rowley, N.curvispinavar.grandiflora (F.Ritter) Donald & Rowley and, N.curvispinavar.garaventae (F.Ritter) Donald & Rowley.

In 1994, Kattermann merged six Ritter’s species into the *E.curvispina* complex: E.curvispinavar.aconcaguensis (F.Ritter) Katt., E.curvispinavar.armata (F.Ritter) Katt., E.curvispinavar.choapensis (F.Ritter) Katt., E.curvispinavar.mutabilis (F.Ritter) Katt., E.curvispinavar.robusta (F.Ritter) Katt. and E.curvispinavar.tuberisulcata (Jacobi) Katt. (=P.horridusvar.horridus F.Ritter). Furthermore, in 2006, Hunt et al. recognized only two of F. Ritter’s species—*Pyrrhocactusarmatus* F.Ritter and *P.marksiana* F.Ritter—as subspecies of *E.curvispina* and synonymized 11 Ritter´s taxa in *E.curvispina*. However, DNA-based phylogenetic analyses supported the exclusion of *E.armata*, *E.marksiana*, and E.marksianavar.lissocarpa from the complex (Guerrero et al. 2019b).

A recent study by Villalobos-Barrantes et al. (2022) included the nine species of *Horridocactus* provided new evidence by incorporating new locations and a new population scale molecular dataset (12 new pairs of nuclear microsatellites, SSR). This significantly enhanced our understanding of the evolutionary relationships among the species of the EriosycesectionHorridocactus. Despite this, no nomenclatural changes were proposed to update the species taxonomy following the genetic analyses. Our objectives were to incorporate more populations ascribed to the *E.curvispina* complex into a DNA-based phylogenetic hypothesis of *Eriosyce*, to evaluate its taxonomic identity, update its taxonomic treatment, and formalize nomenclature changes according to densely sampled phylogenetic inferences based on four plastids and a nuclear molecular marker, and finally, evaluate the conservation status of the delimited species based on IUCN criteria (2017).

## ﻿Materials and methods

### ﻿Plant material and DNA extractions

**Figure 1.**
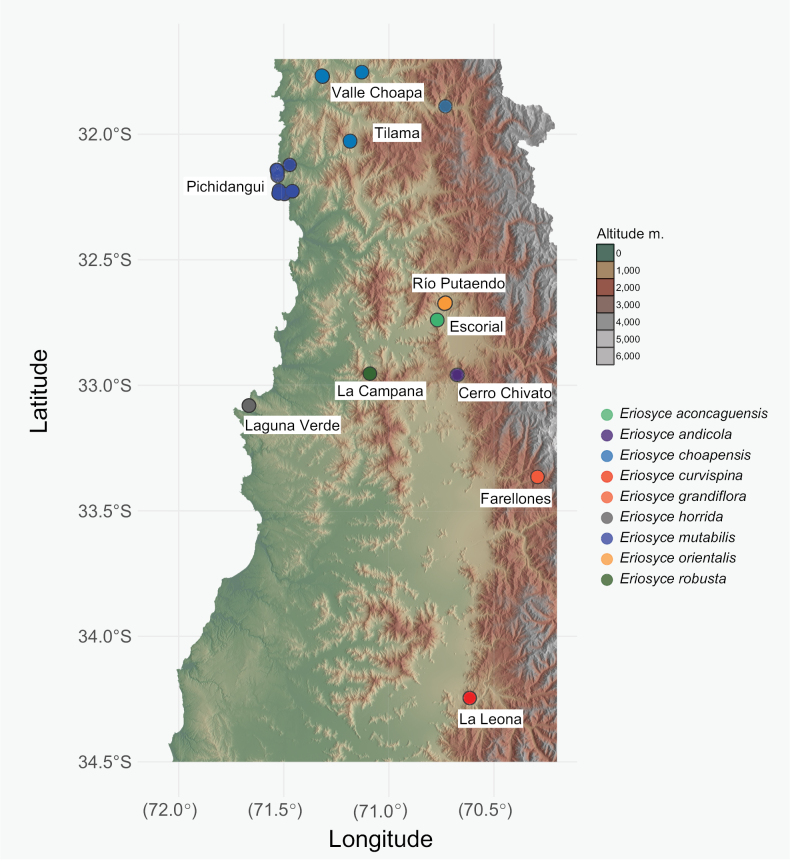
Locations of samples used in the study ascribed to the *Eriosycecurvispina* complex included in phylogenetic inferences.

We examined specimens from the *E.curvispina* complex, including E.curvispinavar.aconcaguensis, E.curvispinavar.choapensis, E.curvispinavar.mutabilis, E.curvispinavar.robusta, and E.curvispinavar.tuberisulcata, as proposed by Kattermann (1994), Hunt et al. (2006), and Guerrero et al. (2019b). Our study also revisited taxa previously included within the *E.curvispina* complex, such as *E.armata*, *E.marksiana*, and E.marksianavar.lissocarpa. The sampling sites (Fig. 1), were chosen to align with the historical localities of populations associated with the *E.curvispina* complex, as described by Ritter (1980) and Kattermann (1994). We included specimens from the type locality of *E.curvispina* near the Cachapoal River at Cerro La Leona (Bertero 1829; Kattermann 1994). Furthermore, we incorporated the nine species of the Horridocactus section into a comprehensive dataset of *Eriosyce* species from all sections. Samples were obtained from individuals spaced at least 30 m apart. A total of 105 samples, represented by 535 sequences (105 samples × 5 markers), were utilized. We incorporated 22 new samples, each sequenced for five markers, thereby contributing with 110 new sequences. This complements sequences previously published by our team in Guerrero et al. (2019b) and Villalobos-Barrantes et al. (2022). We collected plant fresh tissue from roots or flowers mainly and kept them in CTAB-NaCl buffer (2%:22%) to transport to the laboratory and store at -80 °C until the extraction.

For DNA extraction we used 40–50 mg of root or flower tissue that first was pulverized to a fine power using an automatic homogenizer and then total DNA was extracted using DNeasy Plant Kit (Qiagen, Valencia, California, USA). For the phylogenetic analysis, we amplified three noncoding chloroplast markers (*rpl32-trnL*, *trnH-psbA*, *trnL-trnF*), one plastid gene (*ycf1*), and one nuclear gene (PHYC) following the protocol described on Guerrero et al. (2019b). PCR products were checked on 1% agarose gels and then sent to Macrogen (Seoul, Korea) for sequencing in both directions. The DNA datasets can be found at the following link: https://github.com/pabloguerrero-cmd/E_curvispina, and the GenBank numbers of unpublished sequence data can be found in Suppl. material 1.

### ﻿Phylogenetic inferences

A matrix of 105 samples, concatenated from five markers, was assembled, and edited using the program Geneious Prime 2023.1.2 (Biomatters Ltd.). Sequences for each marker were automatically aligned using Muscle and then checked manually. The outgroup consists of 6 species, most from the core Notocacteae. Each marker was aligned separately and then concatenated. A microsatellite region in the *ycf1* dataset was excluded (450 bp) because of ambiguous alignment in this region. Best partitions and molecular models were evaluated using PartitionFinder v.2.1.1 as described in Guerrero et al. (2019b).

Bayesian inferences of the concatenated matrix were performed using Mr.Bayes v3.2.7 (Ronquist et al. 2012) using unlinked rate heterogeneity, based frequencies, and substitution rates across partitions. Bayesian ran for 30 million generations across four independent runs with four chains each, sampling every 1000 generations. The best models were GTR+G for *rpl32-trnL* and *trnH-psbA* and GTR+G+I for the rest of the markers. Convergence was monitored using the standard deviation of split frequencies, and when this value stabilized below 0.01, it was considered a strong indication of convergence. The associated likelihood values, effective sample size (ESS) values, and burn-in values of the different runs were verified with the program Tracer v1.7.1 (Rambaut et al. 2018). Trees were visualized using software FigTree v1.4.4 (Rambaut 2016). Maximum likelihood (ML) analyses of the concatenated matrix were also performed using the program raxmlGUI 2.0 v.2.0.6 (Edler et al. 2021). The search for an optimal ML tree run combined with a rapid bootstrap analysis based on 100 trees and 1000 replicates.

### ﻿Morphological characters

To establish morphological differences between species and build morphological descriptions and an identification key, we used the following diagnostic characters and their (discrete and quantitative) states: stems (habit, size, color); roots (fascicular/ tuberous, size); ribs (shape, size, number); areoles (shape, indumentum, distance, size, color); spines (shape, size, number, color); flowers (pericarpel and hypantium: size, color, indumentum; perianth segments: shape, color; ovary: shape, size; style: size, color); fruit (shape, color, size); seeds (shape, size; testa: color, surface).

### ﻿Extinction risk assessment

We evaluated the extinction risk of each species by applying the IUCN Red List Categories and Criteria (version 3.1, IUCN 2017). This involved calculating the species’ extent of occurrence (EOO) using the spatial tools provided by Google Earth. Additionally, we systematically documented threats based on empirical evidence gathered during fieldwork. The assessments followed a structured approach where the known distribution ranges were analyzed in accordance with Criterion B of the IUCN guidelines. This criterion focuses on geographic range size, the degree of fragmentation, and the level of decline or fluctuation in population size, range, or habitat quality, enabling a comprehensive evaluation of the species’ risk of extinction.

## ﻿Results

The alignment encompasses a total of 4841 nucleotides across 105 individuals, with informative sites varying for each marker, amounting to 2440 for the complete matrix and 1958 for the ingroup (Table 1). The concatenated matrix, which amalgamates all loci, has a total length of 4841 base pairs, with 1565 variable characters within the ingroup, 1754 total variable characters, and 747 parsimony-informative characters. Specifically, the plastid non-coding marker *rpl32-trnL* contributed with 1354 bp (36% of variable sites), *trnL-trnF* contributed with 1084 bp (11%), and *trnH-psbA* contributed with 439 bp (3%). Meanwhile, the plastid gene *ycf1* contributed with 930 bp (24%), and the nuclear gene PHYC contributed with 1034 bp, being the locus with 25% of variable sites.

**Table 1.** Statistics for the 105-sample DNA sequence alignments.

Locus	Total length	Ingroup, variable characters	Total variable characters	Parsimony-informative characters	Ingroup coverage (%)	Outgroup coverage (%)	% variability
*rpl32-trnL*	1354	594	640	286	93	11	36
*trnL-trnF*	1084	161	195	91	83	23	11
*trnH-psbA*	439	36	55	27	65	18	3
*ycf1*	930	341	417	223	82	40	24
PHYC	1034	433	447	120	97	7	25
Concatenated matrix	4841	1565	1754	747			

**Figure 2.**
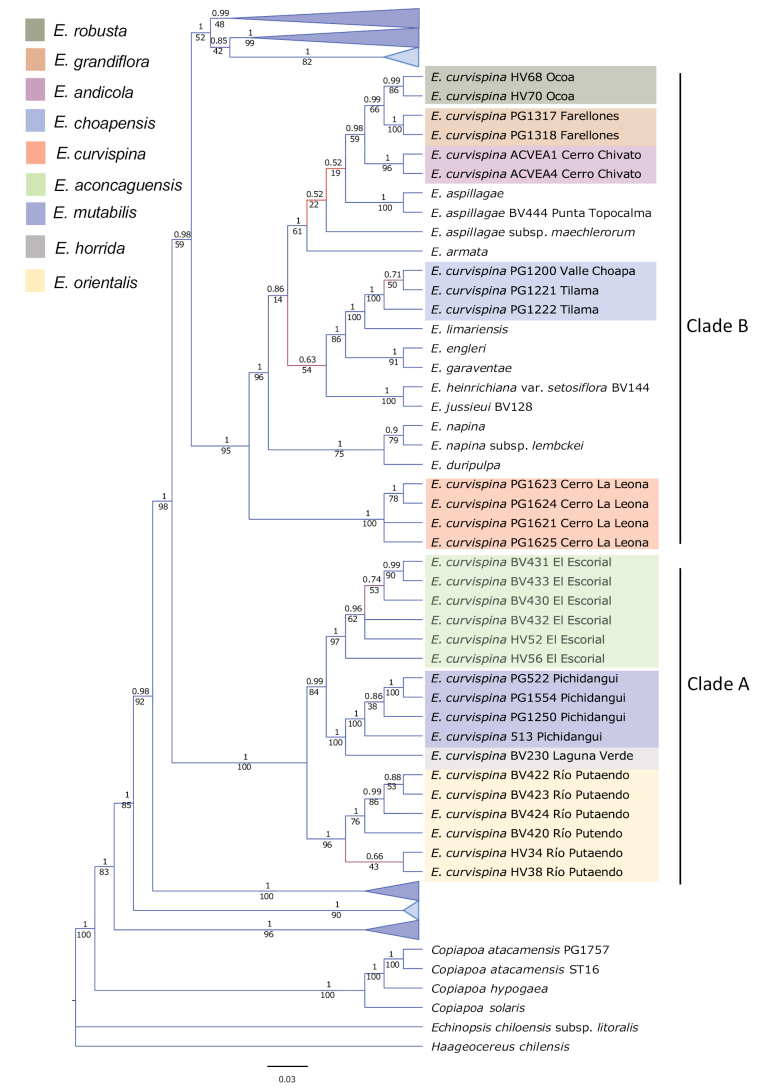
Phylogenetic position of putative members of the *Eriosycecurvispina* species complex. All sections of *Eriosyce* are collapsed, except for the EriosycesectionHorridocactus.

The molecular variation in coding regions is low compared with non-coding regions in general, but all this information was considered in the phylogenetic reconstruction by the Bayesian analysis. A well-supported phylogenetic tree was obtained, indicating nine different lineages of the *E.curvispina* complex (Fig. 2). Two strongly supported clades (A and B) were recovered for the EriosycesectionHorridocactus: Clade A harbors four independent lineages previously assigned to the *E.curvispina* complex between the latitudes of 32°S and 33°S: The six accessions from the Río Putaendo (HV 34, HV 38, BV 420, BV 422, BC 423, BV 424) were placed as sister to the rest of Clade A. The six accessions from El Escorial (HV 52, HV 56, BV 430, BV 431, BV 432, BV 433) were placed as sister to the four accessions from Pichidangui (PG 513, PG 522, PG 554, and PG 1250) and one from Laguna Verde (BV 230).

Clade B is home to several putative members of the *E.curvispina* complex, which comprises four distinct lineages. Firstly, there is a group of four accessions (PG 1621, PG 1623, PG 1624, PG 1625) collected at the type locality of *E.curvispina* in Cerro La Leona, east of Rancagua at 34°S. This group forms a well-supported subclade. Secondly, there is a robust group comprising three specimens, also identified as members of the *E.curvispina* complex, that were collected from various localities in the hills north of the latitude of 32°S. These specimens include Valle Choapa (PG 1200) and Tilama (PG 1221 and PG 1222). This group is placed sister to *E.limariensis* (F.Ritter) Katt. Finally, there is a subclade that harbors accessions from Farellones, east of Santiago (PG 1317, PG 1318), Cerro Chivato (ACVEA 1, ACVEA 2), and Ocoa (HV 68, HV 70), all located at 33°S. These accessions form a strongly supported group.

The EriosycesectionHorridocactus contains several taxa that are scattered across various branches. The northernmost species in this section form a subclade that includes *E.napina* (Phil.) Katt, E.napinavar.lembckei Katt., *E.duripulpa* (F.Ritter) P.C.Guerrero & Helmut Walter. This subclade is followed by a subclade consisting of *E.armata* (F.Ritter) P.C.Guerrero & Helmut Walter from the hills southwest of Santiago, as well as the two southern taxa *E.aspillagae* (F. Ritter) Katt. and E.aspillagaesubsp.maechlerorum Helmut Walter (located at 34'30°–36°S). Additionally, *E.garaventae* (F.Ritter) Katt. and *E.engleri* (F.Ritter) Katt., both found in the high coastal mountains at 33°S, form a small subclade. Finally, the sister pair E.heinrichianavar.setosiflora (F.Ritter) Katt. and *E.jussieui* (Monville ex Salm-Dyck) P.C.Guerrero & Helmut Walter, which occur around the latitude of 30°S, also form a small subclade.

## ﻿Discussion

This incorporation of accessions from previously unexplored populations in this study has substantially deepened our comprehension of the *E.curvispina* complex within the EriosycesectionHorridocactus, leading to a re-evaluation of its taxonomic classification (Guerrero et al. 2019b; Villalobos-Barrantes et al. 2022; Walter and Guerrero 2022). Phylogenetic assessments based on comprehensive sampling reveal that the presumed monophyletic nature of the *E.curvispina* complex is not supported, as certain populations are aligned with different genetic lineages. Additionally, the inclusion of samples from regions such as Cerro Leona in the Province of Cachapoal and in Putaendo in the Province of San Felipe de Aconcagua has prompted significant alterations to the previously established main clades of this complex (Villalobos-Barrantes et al. 2022). While most internal nodes in the phylogenetic tree received strong support, some exhibit only moderate support. To enhance the resolution of these nodes, the application of genomic data is recommended.

In our study, we conducted a focused examination of four accessions from Bertero’s (1829) original location “Cachapoal, Chile”. This locality is situated approximately 100 km south of Santiago and contrasts with F. Ritter’s (1980) type locality “Department Santiago, between 1000 and 2000 m” for his *Pyrrhocactuscurvispinus*. Our phylogenetic analyses reveal that the samples from Bertero’s locality form a distinct clade, separated from the two accessions collected from east Santiago (Ritter’s reported locality for *P.curvispinus*) and other subspecies proposed by Kattermann (1994). These findings support a nomenclatural reevaluation, as the Farellones specimens and Kattermann’s subspecies cannot be accurately classified under the epithet ‘*curvispinus*.’ Given that the Farellones specimens’ morphology and geographic location align more closely with *Pyrrhocactusgrandiflora*, as described by Ritter from ‘Cerro Ramón, east of Santiago, above 2000 m,’ we propose adopting this name (updating in a new combination) for the Farellones populations to maintain nomenclatural continuity without the need for new taxon names. Our results prompt a significant reorganization of the taxa previously included in the *E.curvispina* complex, ensuring that the nomenclature accurately reflects the phylogenetic relationships and geographical distributions.

Our phylogenetic inference aligns with the study by Villalobos-Barrantes et al. (2022) and provides further insight into the evolutionary relationships within section Horridocactus, revealing great complexity in species diversity and their geographic patterns. In that study, at the population level, SSR data revealed substantial genetic divergence even among closely related species, as seen in the case of the Ocoa population (*E.robusta*) and its closest relatives located in Farellones (*E.grandiflora*) and Cerro Chivato (*E.andicola*). High genetic divergence supports their recognition at species rank together with some morphological differences (see section taxonomic treatment and key). Also, the accessions from Tilama and Valle Choapa are supported to be species in their own right (*E.choapensis*) as they are not grouped with other putative members of the *E.curvispina* complex. Furthermore, the populations present low genetic differences inferred from SSR analyses (Villalobos-Barrantes et al. 2022). Some populations distributed in similar habitats can present substantial population genetic divergence, such as the population from Pichidangui (*E.mutabilis*), compared to the southern population located in Laguna Verde (*E.horrida*) (Villalobos-Barrantes et al. 2022), supporting their recognition as a species in its own. An interesting pair of populations are those from Escorial (*E.aconcaguensis*) and the Putaendo river (*E.orientalis*), they were grouped in different branches in the phylogenetic tree. However, despite the limited morphological differences, we developed a morphological key that circumscribes monophyletic species, according to the results of the phylogenetic analyses, accompanied by detailed descriptions of these taxa. This highlights the importance of confirming previously described morpho-species using molecular data.

The new molecular-based classification of EriosycesectionHorridocactus demands the search for diagnostic morphological characters to complement the results obtained with the DNA-based phylogenetic tree. This task is challenging because these species possess few distinguishing features, which has led to past taxonomic synonymization. The historically controversial phylogenetic placement of the large *E.curvispina* complex suggests a combination of processes that have led to the current diversity, with species exhibiting similar morphologies but distinct evolutionary trajectories. The previous concept of *E.curvispina* included a wide distribution of its taxonomic entities, from near sea level to above 2000 m.a.s.l. elevation in the Andes, and between the latitudes 30°S and 36°S. The segregation of the *E.curvispina* complex implies a smaller distribution area for the species delimited here; this has consequences for the extinction risk assessment of taxa.

## ﻿Conclusion

**Figure 3.**
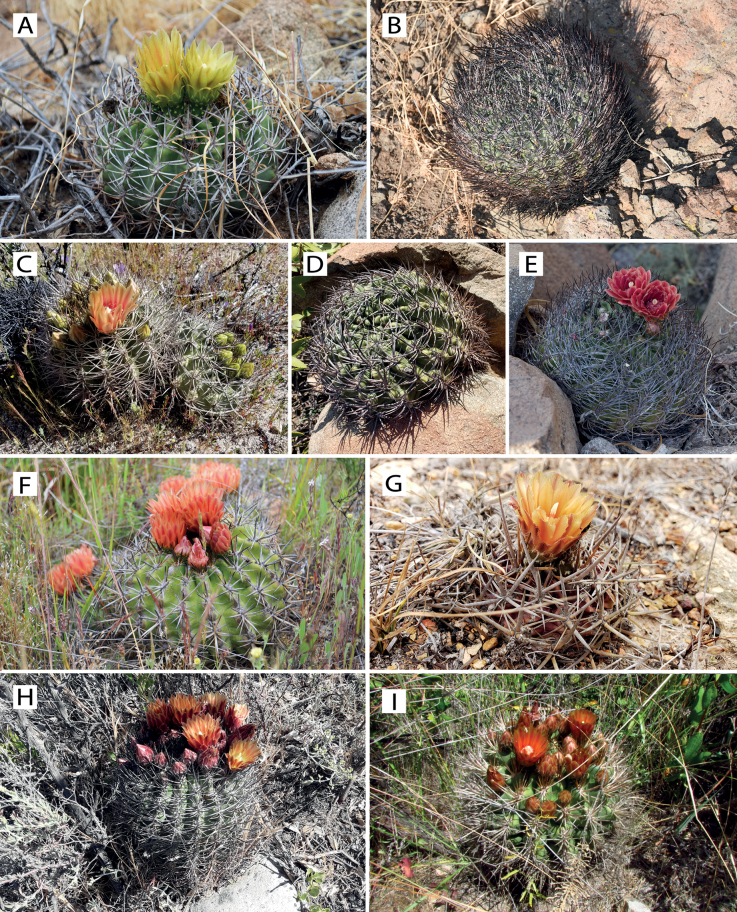
Species of *Eriosycecurvispina* complex **A***E.aconcaguensis***B***E.andicola***C***E.choapensis***D***E.curvispina***E***E.grandiflora***F***E.horrida***G***E.mutabilis***H***E.orientalis***I***E.robusta*. Photographs: Arón Cádiz-Véliz (**A, B, F**), Pablo Guerrero (**C, D, G**), Joaquín Keymer (**E**), Heidy Villalobos-Barrantes (**H**), Griselle Guerrero (**I**).

The phylogenetic analysis of the *E.curvispina* complex, together with the phylogeographic analyses by Villalobos-Barrantes et al. (2022) revealed the presence of nine distinct lineages within the complex (Fig. 3). Two major clades, Clade A and Clade B, were strongly supported. Clade A harbored four independent lineages found between the latitudes of 32°S and 33°S (Río Putaendo, El Escorial, Pichidangui, and Laguna Verde). Clade B comprised four lineages previously considered within the *Eriosycecurvispina* complex, such as the accessions from Cerro La Leona, Valle Choapa, Tilama, Farellones, Cerro Chivato and Ocoa. These results challenge the monophyly of previously defined species complexes within *Eriosyce*, including *E.curvispina*, and support the need to revise these complexes taxonomically. Additionally, our results have implications for the conservation status of the species, which we reassess according to the IUCN guidelines.

### ﻿Taxonomic key to the EriosycesectionHorridocactus

**Table:** 

1	Perianth segments narrowly lanceolate	**2**
–	Perianth segments broad, often spathulate with a short tip	**14**
2	Stems 2–10 cm diameter, grey-green to grey-brown, often pruinose, fruits elongated, perianth remnant attachment area small; taproots always present	**3**
–	Stems 10–20 cm., usually green, never pruinose; fruits short, ovoid to barrel-shape, perianth remnant attachment area large; roots various	**5**
3	Ribs dissolved into tubercles arranged in parastichies; spines very short	**4**
–	Ribs arranged in orthostichies; spines significantly longer	***E.jussieui***
4	Stems never elongating, tubercles large; spines black; pericapel and hypanthium covered with brownish wool and black bristles	***E.napina***
–	Stems extending with age; tubercles small, spines ranging from horn-colored to white; flowers covered with white wool and white bristles	***E.duripulpa***
5	Stems usually not branching	**6**
–	Stems with a tendency towards basal branching	**13**
6	Stems often elongating, spines numerous, often obscuring the stem	**7**
–	Stems subglobose to globose, with fewer spines	**8**
7	Spines long, from white to yellowish, dark-tipped, turning gray with age; floral bristles few, only near hypanthium rim	***E.engleri***
–	Spines shorter, yellow, not turning with age; bristles abundant, covering the hypanthium	***E.garaventae***
8	Roots fascicular from short taproots	**9**
–	Roots fascicular (with the exception of *E.aspillagae*)	**13**
9	Stems up to 15 cm in diameter; ovary substantially elongated; spines up to 4 cm; taproot thick	***E.limariensis***
–	Stems to 10 cm; ovary shorter; spines 1–2 cm; taproot thin	***E.choapensis***
10	Stems 10–20 cm in diameter; ribs 16–24; areoles 1–2 cm long, 1–2 cm apart	**11**
–	Stems 8–12 cm in diameter; ribs 14–16; areoles smaller of 6 × 4 mm and 6 mm apart; spines 1–2.5 cm; flowers 5–5.5 cm	***E.curvispina***
11	Ribs 1.5–2 cm high; areoles 6–10- × 5 mm; pericarpel green	**12**
–	Ribs to 2.5(-3) cm high; areoles larger, 7–20 × 5–8 mm; pericarpel reddish-grown	***E.robusta***
12	Spines numerous, finely acicular with lower portion whitish and upper one brownish-reddish, banded; flowers 5 cm; style white; seed large, 1.5 × 1 mm	***E.andicola***
–	Spines less numerous, stout, blackish-brown, not bicolored nor banded; flowers up to seven cm; style red; seed small, 1 × 0.7 mm	***E.grandiflora***
13	Fruit dry, dehiscing by partial circumscissile splitting; spines finely acicular; ribs broad and low	***E.aspillagae***
–	Fruit-wall fleshy when ripe, dehiscence not as above; spines thicker; ribs steep and narrower	***E.armata***
14	Stems consistently green; spines acicular, curved upwards	**15**
–	Stems either green or brownish; spines thicker and usually straight	**16**
15	Ribs 1.5 cm high; areoles 1.5 cm apart; yellow funnel-form flowers with inner perianth segments lemon yellow without a mid-stripe	***E.aconcaguensis***
–	Ribs 1 cm high; areoles 0.5–0.7 cm apart; pericarpel and hypanthium tube dark violet; exterior perianth segments purple, inner ones pale yellow with a red mid-stripe	***E.orientalis***
16	Stems green,10–20 cm diameter, non-branching; spines 1–2.5 cm, very thick; perianth segments reddish with a dark purple mid-stripe	***E.horrida***
–	Stems often brownish, often branching, 10–15 cm diameter, spines stout 1–5 cm, perianth segments pale yellowish often with a red midstripe	***E.mutabilis***

## ﻿Taxonomic treatment

### ﻿1. Eriosyce aconcaguensis

Taxon classificationPlantaeCaryophyllalesCactaceae

(F.Ritter) P.C.Guerrero & Helmut Walter comb. nov.

E2112A62-BAAD-5D4A-A5A9-002037320FF9

urn:lsid:ipni.org:names:77334726-1

Pyrrhocactus aconcaguensis F. Ritter, Succulenta (NL) 9: 108. 1960. Basionym.
≡ Pyrrhocactus aconcaguensis F.Ritter in Succulenta (Netherlands) 1960: 108. 1960 syn. sec. Tropicos ≡ Horridocactusaconcaguensis (F.Ritter) Backeb., Cactaceae 6: 3791. 1962 syn. sec. Tropicos ≡ Eriosycecurvispinavar.aconcaguensis (F.Ritter) Katt., Eriosyce (Cactac.) Gen. Revis. Ampl. 1: 117. 1994 syn. sec. Kew WCVP (2019) ≡ Neoporteriacurvispinavar.aconcaguensis (F.Ritter) Donald & G.D.Rowley in Cact. Succ. J. Gr. Brit. 28: 55. 1966 syn. sec. Kew WCVP (2019) ≡ Pyrrhocactushorridusvar.aconcaguensis (F.Ritter) F.Ritter, Kakteen Südamerika 3: 948. 1980 syn. sec. Kew WCVP (2019) ≡ Pyrrhocactuscurvispinusvar.felipensis F.Ritter, Kakteen Südamerika 3: 932: 1980 (nom. inval. Art. 34.1, 37.1). In our text, “syn. sec.” refers to the source that assigns a synonym to the concept of either the accepted name or one of its homotypic synonyms *sensu*Korotkova et al. (2021).

#### Type.

Chile, Southern America, Valparaíso Region, Catemu, Chagres, June 1955, *F. Ritter 542* (Holotype: U 0249247, digital image!). (Image available at https://bioportal.naturalis.nl/en/specimen/U__0249247).

#### Iconography.

F. Ritter, Succulenta (NL) 9: 108. 1960 (as *Pyrrhocactusaconcaguensis*); A. Hoffmann & H.E. Walter, Cact. Fl. Sylv. Chile 2^nd^ Ed., Lam. 71a (as Eriosycecurvispinavar.aconcaguensis) 2004.

#### Morphological notes.

Stems globose, 8–12 cm. diameter, sometimes elongating with age. Roots fascicular. Ribs 17–21, 1.5 cm high, obtuse. Areoles 1.5 × 0.6 cm., 1.0–1.5 cm apart. Spines grey, thickly aciculate, mostly straight, or somewhat curved; radial ones 7–12, 1–3 cm long, central ones 4–6, to 4 cm long. Flowers 4–5 cm, funnel-form; pericarpel green, not much elongated, bract-scales small, axils with inconspicuous white hairs; nectary tubular; style white with 14 whitish stigma-lobes; perianth segments yellow, usually with a faint reddish mid-stripe, 0.6–1.2 cm broad. Fruits barrel-form, 1.5–2.5 cm long, reddish, bract-scales as for the pericarpel; perianth remnant attachment area wide, basal pore large. Seeds blackish, 1.2×1 mm; testa finely tuberculate, coarsely ribbed; hilum oval; micropyle in a groove.

#### Distribution.

Endemic species occurring in the middle Río Aconcagua Valley, between Llay-llay and San Felipe (32°S, 70°W) at elevations generally between 500 and 700 m.

#### Conservation status.

The extent of occurrence (EOO) of *E.aconcaguensis* is estimated to be less than 100 km^2^, with fewer than 5 known localities. The species range is severely fragmented, and there has been a decrease in population size and the number of localities due to landscape anthropization. Additionally, there has been a loss of habitat quality due to the expansion of the agricultural and mining industry. The population from the type locality at Chagres (Llay-llay) is likely extinct due to the replacement of xerophytic vegetation by extensive cultivation of avocado trees on slopes between Llay-llay and San Felipe. Therefore, we propose to classify the species as Critically Endangered (CR) according to the criteria (2017) B1ab(i,iii,iv).

### ﻿2. Eriosyce andicola

Taxon classificationPlantaeCaryophyllalesCactaceae

(F.Ritter) P.C.Guerrero & Helmut Walter comb. nov.

D566365F-CDCD-5F41-AA0E-7932F02870F9

urn:lsid:ipni.org:names:77334727-1

Horridocactus andicola F. Ritter, Succulenta (NL) 7: 97. 1959. Basionym.
≡ Pyrrhocactus andicola (F.Ritter) F.Ritter in Succulenta (Netherlands) 10: 131. 1959 syn. sec. Hunt (1999) ≡ Neoporteriacurvispinavar.andicola (F.Ritter) Donald & G.D.Rowley in Cact. Succ. J. Gr. Brit. 28: 55. 1966 syn. sec. Kew WCVP (2019)
= Horridocactus andicola var. descendens F.Ritter in Succulenta (Netherlands) 7: 97. 1959 syn. sec. Kew WCVP (2019) ≡ Neoporteriacurvispinaf.descendens (F.Ritter) Donald & G.D.Rowley in Cact. Succ. J. Gr. Brit. 28: 55. 1966 syn. sec. Kew WCVP (2019)
= Horridocactus andicola var. robustus F.Ritter in Succulenta (Netherlands) 7: 97. 1959 syn. sec. Kew WCVP (2019) ≡ Pyrrhocactusandicolavar.robustus (F.Ritter) F.Ritter in Succulenta (Netherlands) 1959: 131. 1959 syn. sec. Kew WCVP (2019) ≡ Pyrrhocactusandicolavar.mollensis F. Ritter, Succulenta (NL) 10: 131. 1959.

#### Type.

Chile, Southern America, Valparaíso Region, Cerro Chivato between Santiago and Los Andes, May 1955, *F. Ritter 468* (Holotype: U 0249335, digital image!).

(Image available at https://bioportal.naturalis.nl/en/specimen/U__0249335).

#### Iconography.

F. Ritter, Kakt. Sudam. 3: 1980 (as Pyrhocactusandicolavar.robustus); A.E. Hoffmann, Cact. Fl. Sylv. Chile, Lamina 53 a. 1989 (as Neoporteriacurvispinavar.andicola).

#### Morphological notes.

Stems globose, more than 20 cm high with age, apex spiny, 12–16 cm diameter, not branching. Roots fasciculate. Ribs 16–24 cm, 10–15 mm high, notched and somewhat chinned below areoles. Areoles 7–12 mm long and 5–7 mm broad, 1 cm apart. Spines finely aciculate, lower half whitish, upper ones brownish-reddish, dark or light banded; radial spines 10–14, 2–3 cm long, central ones 4–7 cm, somewhat curved upward, 3–4 cm long. Flowers ca. 5 cm, funnel-form; pericarpel and tube green, interior white, bract scales tiny, yellowish, axils with white inconspicuous thin bristles and scarce wool; perianth segments 0.7–1cm broad, spathulate with short tips; lemon- to olive-yellow, often with a narrow pale purple central stripe; style basally widened, whitish; stigma-lobes whitish; filaments whitish; nectary tubular; ovary elongated. Fruit pale red, 1.5 cm, barrel-shape; bract-scales inconspicuous; basal pore large; perianth remnant attachment wide. Seeds ca. 1.5 mm, short ovoid; testa black, opaque, finely tuberculate; ventrally strongly bulged, notched below hilum; hilum shortly ovoid, position ventral.

#### Distribution.

Endemic species occurring between the Rio Molles (30°S, 70°W) and Cerro Chivato (33°S, 70°W) at altitudes between 1000 and 2000 m.

#### Conservation status.

The Extent of Occurrence (EOO) of *E.andicola* is estimated to be < 20.000 km^2^, with its populations severely fragmented due to landscape anthropization. There has been an estimated decrease in population size and the number of localities, as well as a loss of habitat quality due to mining activities, stump removal, and livestock. We propose to classify the species as Vulnerable (VU) according to criteria B1ab(i,iii,iv).

### ﻿3. Eriosyce choapensis

Taxon classificationPlantaeCaryophyllalesCactaceae

(F.Ritter) P.C.Guerrero & Helmut Walter comb. nov.

8F056A95-604D-5FCA-B283-2DAEEA1110B7

urn:lsid:ipni.org:names:77334728-1

Pyrrhocactus choapensis F. F.Ritter, Succulenta (NL)12: 133. 1960. Basionym.
≡ Pyrrhocactus choapensis F.Ritter in Succulenta (Netherlands) 1960: 133. 1960 syn. sec. Tropicos ≡ Horridocactuschoapensis (F.Ritter) Backeb., Cactaceae 6: 3793. 1962 syn. sec. Tropicos ≡ Neoporteriachoapensis (F.Ritter) Donald & G.D.Rowley in Cact. Succ. J. Gr. Brit. 28: 55. 1966 syn. sec. Tropicos ≡ Eriosycecurvispinavar.choapensis (F.Ritter) Katt., Eriosyce (Cactac.) Gen. Revis. Ampl. 1: 117. 1994 syn. sec. Kew WCVP (2019).

#### Type.

Chile, Southern America, Coquimbo Region, Choapa, Illapel, May 1954, *F. Ritter 238* (Holotype: ZSS 005073, Isotypes: SGO, ZSS).

#### Iconography.

F. Ritter, Kakt. Sudam. 3, 1980.

#### Morphological notes.

Stems subglobose, dark green, often burnt brownish, 5–10 cm diameter Roots fascicular with a short thin taproot. Ribs 15–22, obtuse, deeply notched, 1.0–1.5 cm high. Areoles long-oval, 1cm, 1 cm apart. Spines are thin, acicular, grey-brown, short, 1–2 cm; radial ones 8–10, somewhat curved; central ones 4–9, somewhat thicker. Flowers funnel-form, 3.5–4.5 cm; pericarpel and hypanthium bract scales tiny, red, axils with white inconspicuous wool; nectary isodiametric; style pinkish, stigma lobes yellow; perianth segments narrow lanceolate, pale yellow with a faint purple mid-stripe. Fruits 1.5–2 cm, barrel-form, indumentum as for the flower; perianth remnant attachment area wide, basal pore large. Seeds small, 0.8–9 mm, round oval, blackish brown; testa cells arranged in ribs, medium-domed; hilum oval, positioned nearly basal, micropyle in a groove.

#### Distribution.

Endemic species occurring strictly inland, from Illapel (31°S, 71°W) to Tilama (32°S, 71°W) at elevations between 400 and 1200 m.

#### Conservation status.

The Extent of Occurrence (EOO) of *E.choapensis* is estimated at < 5000 km^2^, with its populations severely fragmented due to landscape anthropization. There has been an estimated decrease in population size and the number of localities, as well as a loss of habitat quality due to mining activities, stump removal, and livestock. We propose to classify the species as Endangered (EN) according to criteria B1ab(i,iii,iv).

### ﻿4. Eriosyce curvispina

Taxon classificationPlantaeCaryophyllalesCactaceae

(Bertero ex Colla) Katt.

C682E3CA-6143-5F91-9B81-4B36442F8D5C

Cactus curvispinus Bertero ex Colla in Mem. Reale Accad. Sci. Torino 37: 76. 1834 syn. sec. Hunt et al. (2006). Basyonym.
≡ Cactus curvispinus Bertero ex Colla in Mem. Reale Accad. Sci. Torino 37: 76. 1834 syn. sec. Hunt et al. (2006) ≡ Echinocactuscurvispinus (Bertero ex Colla) Gay, Fl. Chil. 3: 16. 1848 syn. sec. Kew WCVP (2019) ≡ Malacocarpuscurvispinus (Bertero ex Colla) Britton & Rose, Cactaceae 3: 203. 1922 syn. sec. Kew WCVP (2019) ≡ Pyrrhocactuscurvispinus (Bertero ex Colla) A.Berger, Kakteen: 345. 1929 syn. sec. Kew WCVP (2019) ≡ Horridocactuscurvispinus (Bertero ex Colla) Backeb. in Kakteenkunde 1940: 51. 1940 syn. sec. Kew WCVP (2019) ≡ Hildmanniacurvispina (Bertero ex Colla) Kreuz. & Buining in Repert. Spec. Nov. Regni Veg. 50: 207. 1941 syn. sec. Kew WCVP (2019) ≡ Neoporteriacurvispina (Bertero ex Colla) Donald & G.D.Rowley in Cact. Succ. J. Gr. Brit. 28: 55. 1966 syn. sec. Kew WCVP (2019)Neoporteriacurvispina (Bertero ex Colla) Don. & Rowl., Cact. Succ. J. (GB) 28: 55. 1966; Pyrrhocactuscurvispinusvar.mostazalensis F.Ritter, Kakt. Sudam. 3: 932. 1980 (nom. inval., Art. 34.1, 37.1); Pyrrhocactuscurvispinusvar.australis F. Ritter, Kakt. Sudam. 3:932. 1980 (nom. inval., Art. 34.1, 37.1).

#### Type.

Chile, Southern America, O’ Higgins Region, Cachapoal (Locotypus). Neotype (designated by F. Kattermann 1994): Colla, I.c.t. 16.2. [132].

#### Iconography.

F. Kattermann, Succ. Pl. Res. 1, Pl. 6.5 and 6.6. 1994.

#### Morphological notes.

Stems dark green, subglobose to globose, not elongating with age; 8–12 cm diameter Roots fascicular. Ribs 14–16, well pronounced, 1–1.5 cm high, notched below areoles, tubercles chinned. Areoles with short wool, oval, 6 × 4 mm, 6 mm apart. Spines horn-coloured, turning grey, acicular, curved; 10–12 radials, curved sideward, 1–2 cm; 1–4 centrals, strongly curved upward, 2–2.5 cm. Flowers diurnal, funnel-form, 5 cm; pericarpel and hypanthium with small bract scales, axils with inconspicuous wool, upper ones with short fine bristles; perianth segments lanceolate, 6 mm wide, yellowish with a reddish mid-stripe of different width; nectary tubular; style reddish, stigma lobes yellow to pale red; ovary isodiametric to elongate. Fruits covered as for the flower, short barrel-shape, 1.5–2 cm, reddish to red brown; perianth remnant attachment area large, basal pore large; fruit wall thick. Seeds oval, 1.3 × 1.2 mm; testa blackish brownish, evenly tuberculate, not ribbed, cells high domed; hilum narrow oval; position oblique; micropyle in a groove.

#### Distribution.

The endemic species occurs from the Rio Maipo Valley (34°S, 70°W) to the Rio Maule Valley (36°S, 70°W) at strictly inland habitats between 1000 and 2000 m.

#### Conservation status.

The Extent of Occurrence (EOO) of *E.curvispina* is estimated at < 20,000 km^2^, with its populations severely fragmented due to landscape anthropization. There has been an estimated decrease in population size and the number of localities. Additionally, there has been a loss of habitat quality due to urban expansion, mining activities, forest fires, agriculture, livestock, and stump removal. We propose to classify the species as Vulnerable (VU) according to criteria B1ab(i,iii,iv).

### ﻿5. Eriosyce grandiflora

Taxon classificationPlantaeCaryophyllalesCactaceae

(F.Ritter) P.C.Guerrero & Helmut Walter comb. nov.

8CFCAAAA-7E61-5F07-9391-05E252B88EED

urn:lsid:ipni.org:names:77334729-1

Pyrrhocactus grandiflorus F.Ritter, Succulenta (NL) 4: 41. 1960. Basyonym.
≡ Pyrrhocactus grandiflorus F.Ritter in Succulenta (Netherlands) 4: 41. 1960 syn. sec. Kew WCVP (2019) ≡ Horridocactusgrandiflorus (F.Ritter) Backeb., Cactaceae 6: 3796. 1962 syn. sec. Kew WCVP (2019) ≡ Neoporteriacurvispinavar.grandiflora (F.Ritter) Donald & G.D.Rowley in Cact. Succ. J. Gr. Brit. 28: 56. 1966 syn. sec. Kew WCVP (2019) ≡ Pyrrhocactuscurvispinusvar.santiagensis F. Ritter, Kakt. Südamerika 3: 932. 1980 (nom. inval., Art. 34.1, 37.1).

#### Type.

Chile, Southern America, Santiago Region, San Ramón, 2000 m, May 1955, *F. Ritter 469* (Holotype: U 0249323, digital image!). (Image available at https://bioportal.naturalis.nl/en/specimen/U__0249323).

#### Iconography.

F. Ritter, Kakt. Südam. 3, 1980.

#### Morphological notes.

Stems simple, grey-green, subglobose, 10–18 cm diameter Roots fascicular. Ribs many, 21–24, 1–1.5 cm high, notched below areoles, tubercles with chin-like protrusions. Areoles 6 × 10 × 5 mm, up to 1.2 cm apart. Spines are acicular, blackish-brown; radial ones 9–12, straight or somewhat curved upward, 1.5–3 cm; central ones 4–7, curved upward, 2–4 cm. Flowers large, 6–7 cm, pericarpel and hypanthium with small bract scales, axils with very short wool, upper ones with short fine bristles; perianth segments lanceolate, 7–10 mm wide and 3.5–4.5 cm long, red with a darker mid-stripe; nectary tubular; style white, superior portion pale reddish, stigma lobes yellow; ovary isodiametric to somewhat elongate. Fruits 1.5 cm, covered as for the flower, short barrel-shape, 1.5 cm, reddish to red brown; perianth remnant attachment area large, basal pore large; fruit wall thick. Seeds 1 × 0.8 mm; testa blackish brownish, evenly tuberculate, not ribbed, cells medium-domed; hilum oval; position oblique.

#### Distribution.

Endemic species occurring from east of Santiago (33°S, 70°W) to the upper Río Aconcagua Valley (32°S, 70°W) at high altitudes, around 2000 m.

#### Conservation status.

The Extent of Occurrence (EOO) of *E.grandiflora* is estimated at < 5000 km^2^, with presence in 5 or less locations. It is estimated that there will be a decrease in population size and number of localities, loss of habitat quality due to mining activities, opening of roads, livestock and stump removal. We propose to classify the species as Endangered (EN) according to the criteria B1ab(i,iii,iv).

### ﻿6. Eriosyce horrida

Taxon classificationPlantaeCaryophyllalesCactaceae

(Remy ex Gay) P.C.Guerrero & Helmut Walter comb. nov.

2E6D8D4D-EF72-58B8-96D0-582D2AA7A3AD

urn:lsid:ipni.org:names:77334730-1

Echinocactus horridus Remy ex Gay, Fl. Chil. 3: 15. 1848. Basyonym.
≡ Cactus horridus Colla in Mem. Reale Accad. Sci. Torino 37: 76. 1834 syn. sec. Hunt et al. (2006) ≡ Echinocactushorridus Gay, Fl. Chil. 3: 15. 1848 syn. sec. Tropicos ≡ Pyrrhocactushorridus (Colla) Backeb., Kaktus-ABC: 264. 1936 [“1935”] syn. sec. Kew WCVP (2019) ≡ Horridocactushorridus (Colla) Backeb. in Kakteenkunde 1940: 51. 1940 syn. sec. Tropicos ≡ Hildmanniahorrida (Colla) Kreuz. & Buining in Repert. Spec. Nov. Regni Veg. 50: 207. 1941 syn. sec. Kew WCVP (2019) ≡ Neoporteriahorrida (Gay) D.R.Hunt in Bradleya 5: 93. 1987 syn. sec. Kew WCVP (2019) = Echinocactustuberisulcatus Jacobi in Allg. Gartenzeitung 24: 108. 1856 syn. sec. Korotkova et al. (2006) ≡ Malacocarpustuberisulcatus (Jacobi) Britton & Rose, Cactaceae 3: 202–203. 1922 syn. sec. Korotkova et al. (2006) ≡ Pyrrhocactustuberisulcatus (Jacobi) A.Berger, Kakteen: 215. 1929 syn. sec. Korotkova et al. (2006) ≡ Horridocactustuberisulcatus (Jacobi) Y.Itô, Cacti, ed. 2: 80. 1952 syn. sec. Korotkova et al. (2006) ≡ Neoporteriatuberisulcata (Jacobi) Donald & G.D.Rowley in Cact. Succ. J. Gr. Brit. 28: 58. 1966 syn. sec. Korotkova et al. (2006) ≡ Eriosycecurvispinavar.tuberisulcata (Jacobi) Katt., Eriosyce (Cactac.) Gen. Revis. Ampl.1: 117. 1994 syn. sec. Korotkova et al. (2006) ≡ Pyrrhocactusodoriflorus F.Ritter in Succulenta (Netherlands) 1960: 116. 1960 syn. sec. Kew WCVP (2019) ≡ Neochileniaodoriflora (F.Ritter) Backeb., Cactaceae 6: 3778. 1962 syn. sec. Kew WCVP (2019) ≡ Neoporteriahorridavar.odoriflora (F.Ritter) A.E.Hoffm., Cact. Fl. Silvestre Chile: 190. 1989 syn. sec. Kew WCVP (2019).

#### Type.

Chile, Southern America, Valparaíso Region, Valparaíso (Locotypus). Lectotype (designated by F. Kattermann 1994): Bertero (ТО) [171].

#### Iconography.

Kattermann, Succ. Pl. Res. 1, Pl. 7 (5) as “var. horrida”(sic!), and as E.curvispinavar.tuberisulcata). 1994; C. Bacheberg, Das Kakteenlexikon, Abb. 170, 171. 1977 (as *Horridocactustuberisulcatus*).

#### Morphological notes.

Stems green, 10–20 cm diameter Ribs 14–18(-20), ca. 1.5 cm high, deeply notched. Areoles 0.7–1.8 × 0.5–1.0 cm. Spines brown, later grey, usually thick, only slightly curved upward; radials 9–12, 1–2 cm, central ones 4–8, 1.5–3 cm long. Flowers funnel-form, 4–5 cm; pericarpel bract-scales small, axils usually with inconspicuous wool, hypanthium sometimes with tortuous bristles; perianth segments 1–1.2 cm broad with short tips, mostly reddish with a dark purple mid-stripe, sometimes rose with a pale mid-stripe; ovary isodiametric, style reddish, stigma-lobes yellow. Fruits barrel-shape, 1.5 cm, indumentum as for the pericarpel; perianth remnant attachment area wide, basal pore large. Seeds 0.8–1 mm, round oval, opaque, blackish brown; testa finely tuberculate, only slightly ribbed; hilum oval, position ventrally oblique, micropyle in a groove.

#### Distribution.

Endemic species, occurring on low coastal hills between south of Valparaíso (33°S, 71°W) and Papudo (32°S, 71°W).

#### Conservation status.

The Extent of Occurrence (EOO) of *E.horrida* is estimated to be < 5000 km^2^, with its populations severely fragmented by urban areas, forest lands, and electric highways. It is estimated that there will be a decrease in the population size and number of localities, loss of habitat quality due to expansion of the real estate industry, forest fires and stump removal. We propose to classify the species as Endangered (EN) according to the criteria B1ab(i,iii,iv).

### ﻿7. Eriosyce mutabilis

Taxon classificationPlantaeCaryophyllalesCactaceae

(F.Ritter) P.C.Guerrero & Helmut Walter, comb. & stat. nov.

44F62224-2F1E-5521-9917-CCE491E7D451

urn:lsid:ipni.org:names:77334731-1

Pyrrhocactus horridus var. mutabilis F. Ritter, Kakt. Sudam. 3: 946. 1980. Basyionym.
≡ Eriosyce curvispina var. mutabilis (F.Ritter) Katt., Succ. Pl. Res. 1: 117. 1994.

#### Type.

Chile, Southern America, Coquimbo Region, coastal spur N of Los Vilos, December 1955, *F. Ritter 223b* (Holotype: U 0249320, digital image!). (Image available at https://bioportal.naturalis.nl/en/specimen/U__0249320).

#### Iconography.

F. Ritter, Kakt. Sudam. 3, 1980; F. Kattermann, Succ. Pl. Res. 1, 1994.

#### Morphological notes.

Stems green, often burnt brownish, branching, 8–15 cm diameter Ribs ca. 16, 1.2 cm high, deeply notched. Areoles oval, 0.6–1.0 long. Spines brown, later grey, thickly acicular; radials 9–12, 1–3.5 cm, slightly bent; central ones 3–7, 1.5–4 cm long, shorter ones straight, longer ones bent upward. Flowers funnel-form, 3–4 cm; pericarpal and hypantium bract-scales small, axils with inconspicuous wool; perianth segments 1–1.2 cm broad with short tips, pale yellow or brownish yellowish, often with a pale reddish mid-stripe of various width; ovary isodiametric, style reddish, stigma-lobes yellow. Fruits barrel-shape, 1.5 cm, indumentum as for the flower; perianth remnant attachment area wide, basal pore large. Seeds 0.9–1.1 mm, round oval, opaque, brown; testa tuberculate and ribbed; hilum oval, position ventrally oblique, micropyle in a groove.

#### Distribution.

Endemic species, occurring between Los Vilos (31°S, 71°W) and Los Molles (32°S, 71°W), near the coast.

#### Conservation status.

the extent of occurrence (EOO) of *E.mutabilis* is estimated to be less than 100 km^2^, with its populations severely fragmented by population centers and road openings. Additionally, there has been a decrease in population size and the number of localities, as well as a loss of habitat quality due to real estate expansion, forest fires, and destemming. We propose to classify the species as Critically Endangered (CR) according to criteria B1ab(i,iii,iv).

### ﻿8. Eriosyce orientalis

Taxon classificationPlantaeCaryophyllalesCactaceae

(F.Ritter) P.C.Guerrero & Helmut Walter, comb. & stat. nov.

60576066-36EA-5866-A2EE-218C1DE3B943

urn:lsid:ipni.org:names:77334732-1

Pyrrhocactus aconcaguensis var. orientalis F. Ritter, Succulenta (NL) 9: 109. 1960. Basionym.
≡ Horridocactus aconcaguensis var. orientalis (F.Ritter) Backeb., Cactaceae 6: 3791. 1962 syn. sec. Kew WCVP (2019) ≡ Pyrrhocactushorridusvar.orientalis (F.Ritter) F.Ritter, Kakteen Südamerika 3: 949. 1980 syn. sec. Kew WCVP (2019) ≡ Neoporteriacurvispinaf.orientalis (F.Ritter) Donald & G.D.Rowley in Cact. Succ. J. Gr. Brit. 28: 55. 1966 syn. sec. Kew WCVP (2019).

#### Type.

Chile, Southern America, Valparaíso Region, San Felipe, Las Coimas, June 1955, *F. Ritter 542a* (Holotype: U 0249247, digital image!). (Image available at https://bioportal.naturalis.nl/en/specimen/U__0249247); (Isotype: SGO 121636!).

#### Iconography.

H.M. Villalobos-Barrantes et al., Genes, 2022.

#### Morphological notes.

Stems pale green, globose 7–14 cm diameter, globose. Ribs ca. 20, obtuse, 1 cm high; tubercles small, somewhat chinned. Areoles small, 0.5–0.7 × 0.4–0.5 cm, 1 cm apart. Spines 2–4 cm acicular; radial ones 7–9, 0.5–1.5 cm long, radiating, straight to somewhat curved; centrals 3–6, mostly curved upward, 3–4 cm. Flowers 4–5 cm, funnel-form; pericarpel and hypanthium dark purple, with small scales, axils with inconspicuous white wool; perianth segments 6–8 mm wide with a long tip; interior ones pale yellowish, with a broad faint reddish mid-stripe, exterior ones purple; ovary isodiametric; style white. Fruits ca. 1.5 cm, barrel-form; indumentum as for the flower; perianth remnant attachment area wide, basal pore large.

#### Distribution.

Endemic species, occurring around Putaendo (32°S, 70°W) at elevations between 600–1900 m.

#### Conservation status.

It is estimated that the Extent of Occurrence (EOO) of *E.orientalis* is less than 100 km^2^, with its main population restricted to the dry bed of the Putaendo river, severely fragmented by the construction of roads and aggregate extraction centers. A registered population in the Rocín River (Las Tejas Sector) is threatened by mining. There is an estimated decrease in population size and the number of localities, as well as a loss of habitat quality due to the increase in micro-dumps, mining, and vine removal. We propose to classify the species as Critically Endangered (CR) according to criteria B1ab(i,iii,iv).

### ﻿9. Eriosyce robusta

Taxon classificationPlantaeCaryophyllalesCactaceae

(F.Ritter) P.C.Guerrero & Helmut Walter comb. nov.

A4271331-4FAD-58A6-A141-817D77B51567

urn:lsid:ipni.org:names:77334733-1

Pyrrhocactus robustus F. Ritter, Succulenta (NL) 6: 65. 1960. Basyonym.
≡ Pyrrhocactus robustus F.Ritter in Succulenta (Netherlands) 1960: 65. 1960 syn. sec. Tropicos ≡ Pyrrhocactushorridusvar.robustus (F.Ritter) in Kakteen in Südamerika 3: 947 ≡ Neochileniarobusta (F.Ritter) Backeb., Cactaceae 6: 3781. 1962 syn. sec. Tropicos ≡ Eriosycecurvispinavar.robusta (F.Ritter) Katt., Eriosyce (Cactac.) Gen. Revis. Ampl. 1: 117. 1994 syn. sec. Kew WCVP (2019) ≡ Neoporteriatuberisulcatavar.robusta (F.Ritter) Donald & G.D.Rowley in Cact. Succ. J. Gr. Brit. 28: 58. 1966 syn. sec. Kew WCVP (2019) ≡ Neoporteriacurvispinaf.robusta (F.Ritter) Donald & G.D.Rowley in Cact. Succ. J. Gr. Brit. 28: 55. 1966 syn. sec. Kew WCVP (2019) ≡ Pyrrhocactushorridusvar.robustus (F.Ritter) F.Ritter, Kakteen Südamerika 3: 947. 1980 syn. sec. Kew WCVP (2019) = Pyrrhocactusrobustusvar.vegasanus F.Ritter in Succulenta (Netherlands) 1960: 65. 1960 syn. sec. Kew WCVP (2019) ≡ Neochileniarobustavar.vegasana (F.Ritter) Backeb., Cactaceae 6: 3783. 1962 syn. sec. Kew WCVP (2019) ≡ Neoporteriatuberisulcatavar.vegasana (F.Ritter) Donald & G.D.Rowley in Cact. Succ. J. Gr. Brit. 28: 58. 1966 syn. sec. Kew WCVP (2019).

#### Type.

Chile, Southern America, Valparaíso Region, Quillota, Ocoa, *F. Ritter 239a* (Holotype: U 0249318, digital image!). (Image available at https://bioportal.naturalis.nl/en/specimen/U__0249318).

#### Iconography.

F. Ritter, Kakt. Sudam. 3: 1980; A. Hoffmann & H. E. Walter, Cact. Fl. Sylv. Chile 2^nd^ Ed., Lam. 72b (as Eriosycecurvispinavar.robusta) 2004; F. Kattermann, Succ. Pl. Res 1, 1994.

#### Morphological notes.

Stems globose, somewhat elongating with age, 10–20 cm diameter Roots fascicular. Ribs 13–20, broad, obtuse and 1.5–3.0 high, deeply notched, tubercles with a long chin. Areoles 0.7–2 × 0.5–0.8 cm, 1–4 cm apart. Spines mostly stout, gray-brown, the shorter ones mostly straight, the longer ones somewhat curved upward; radial ones 7–12, 1–3 cm long; central ones 1–8, 1–4 cm. Flowers 4–5 cm, funnel-form; pericarpel and hypanthium with small scales, axils with inconspicuous white wool, pericarpel much elongated, brownish-red; perianth segments lanceolate, 0.5–1.0 cm, lower part purple or carmin, superior part pale yellowish; ovary elongated; style whitish, superior part pink. Fruits1–2 × 0.5–1 cm, long barrel-form; indumentum as for the flower; perianth remnant attachment area wide, basal pore large. Seeds 1.2 mm, round ovate, opaque, brownish blackish; testa coarsely tuberculate and ribbed; hilum nearly round, position ventrally oblique, micropyle in a groove.

#### Distribution.

Endemic species occurring in the region around the National Park La Campana (32°S, 71°W).

#### Conservation status.

It is estimated that the Extent of Occurrence (EOO) of *E.robusta* is < 5000 km^2^, with its population severely fragmented by urban centers, electric highways and agro-industrial crops. It is estimated there will be a decrease in the population size and number of localities, loss of habitat quality due to urban expansion and agro-industry, forest fires and stump removal. We propose to classify the species as Endangered (EN) according to criteria B1ab(i,iii,iv).

## Supplementary Material

XML Treatment for Eriosyce aconcaguensis

XML Treatment for Eriosyce andicola

XML Treatment for Eriosyce choapensis

XML Treatment for Eriosyce curvispina

XML Treatment for Eriosyce grandiflora

XML Treatment for Eriosyce horrida

XML Treatment for Eriosyce mutabilis

XML Treatment for Eriosyce orientalis

XML Treatment for Eriosyce robusta

## ﻿Additional information

### Author ORCIDs

Arón Cádiz-Véliz https://orcid.org/0000-0002-7548-1764

Beatriz M. Meriño https://orcid.org/0000-0002-9518-2331

Heidy M. Villalobos-Barrantes https://orcid.org/0000-0003-3657-4439

Pablo C. Guerrero https://orcid.org/0000-0003-1034-1899

### Data availability

All of the data that support the findings of this study are available in the main text or Supplementary Information.

## Supplementary materials

Supplementary material 1 New accessions of taxa used in the phylogenetic analyses, including their laboratory code, population locality, and GenBank numbers Data type docx https://binary.pensoft.net/file/971927This dataset is made available under the Open Database License (http://opendatacommons.org/licenses/odbl/1.0/). The Open Database License (ODbL) is a license agreement intended to allow users to freely share, modify, and use this Dataset while maintaining this same freedom for others, provided that the original source and author(s) are credited.Helmut E. Walter, Arón Cádiz-Véliz, Beatriz M. Meriño, Heidy M. Villalobos-Barrantes, Pablo C. Guerrero
