# Supplementary material for: ﻿Taxonomic dissection based on molecular evidence of the Eriosycecurvispina complex (Cactaceae): identifying nine endemic species from Central Chile
Source: PhytoKeys. 2024 Jan 22;237:117–39. doi: 10.3897/phytokeys.237.107403 (PMC10825970; doi:10.3897/phytokeys.237.107403)
Supplement: Supplementary material 1 — New accessions of taxa used in the phylogenetic analyses, including their laboratory code, population locality, and GenBank numbers [file phytokeys-237-117_article-107403__-s001.docx]

**Supplementary material 1**

Table S1: New accessions of taxa used in the phylogenetic analyses, including their laboratory code, population locality, and GenBank numbers.

| **Taxon** | **Lab code** | **Location** | ***rpL32-trnL*** | ***trnL-trnF*** | ***trnH-psbA*** | ***ycf1*** | **PHYC** |  |
| --- | --- | --- | --- | --- | --- | --- | --- | --- |
| *Copiapoa atacamensis* | PG1757 | La Chimba | OR807063 | OR786375 | OR786397 | OR826355 | OR807085 |  |
|  |  |  |  |  |  |  |  |  |
| *Copiapoa atacamensis* | ST16 | Morro Moreno | OR807064 | OR786376 | OR786398 | OR826356 | OR807086 |  |
|  |  |  |  |  |  |  |  |  |
| *Eriosyce aspillagae* | BV444 | Punta Tococalma | OR807065 | OR786377 | OR786399 | OR826357 | OR807087 |  |
|  |  |  |  |  |  |  |  |  |
| *Eriosyce atroviridis* | BV291 | Quebradita, Freirina | OR807066 | OR786378 | OR786400 | OR826358 | OR807088 |  |
|  |  |  |  |  |  |  |  |  |
| *Eriosyce calderana* | PG1650 | Quebrada El León | OR807067 | OR786379 | OR786401 | OR826359 | OR807089 |  |
|  |  |  |  |  |  |  |  |  |
| *Eriosyce calderana* subsp. *pulchella* | PG1656 | Portofino, sector La Virgen | OR807068 | OR786380 | OR786402 | OR826360 | OR807090 |  |
|  |  |  |  |  |  |  |  |  |
| *Eriosyce curvispina* | ACVEA1 | Cerro Chivato | OR807069 | OR786381 | OR786403 | OR826361 | OR807091 |  |
|  |  |  |  |  |  |  |  |  |
| *Eriosyce curvispina* | ACVEA4 | Cerro Chivato | OR807070 | OR786382 | OR786404 | OR826362 | OR807092 |  |
|  |  |  |  |  |  |  |  |  |
| *Eriosyce curvispina* | BV420 | Putaendo | OR807071 | OR786383 | OR786405 | OR826363 | OR807093 |  |
|  |  |  |  |  |  |  |  |  |
| *Eriosyce curvispina* | BV422 | Putaendo | OR807072 | OR786384 | OR786406 | OR826364 | OR807094 |  |
|  |  |  |  |  |  |  |  |  |
| *Eriosyce curvispina* | BV423 | Putaendo | OR807073 | OR786385 | OR786407 | OR826365 | OR807095 |  |
|  |  |  |  |  |  |  |  |  |
| *Eriosyce curvispina* | BV424 | Putaendo | OR807074 | OR786386 | OR786408 | OR826366 | OR807096 |  |
|  |  |  |  |  |  |  |  |  |
| *Eriosyce curvispina* | BV430 | El Escorial | OR807075 | OR786387 | OR786409 | OR826367 | OR807097 |  |
| *Eriosyce curvispina* | BV431 | El Escorial | OR807076 | OR786388 | OR786410 | OR826368 | OR807098 |  |
| *Eriosyce curvispina* | BV432 | El Escorial | OR807077 | OR786389 | OR786411 | OR826369 | OR807099 |  |
| *Eriosyce curvispina* | BV433 | El Escorial | OR807078 | OR786390 | OR786412 | OR826370 | OR807100 |  |
| *Eriosyce curvispina* | PG1621 | Cerro La Leona | OR807079 | OR786391 | OR786413 | OR826371 | OR807101 |  |
| *Eriosyce curvispina* | PG1623 | Cerro La Leona | OR807080 | OR786392 | OR786414 | OR826372 | OR807102 |  |
| *Eriosyce curvispina* | PG1624 | Cerro La Leona | OR807081 | OR786393 | OR786415 | OR826373 | OR807103 |  |
| *Eriosyce curvispina* | PG1625 | Cerro La Leona | OR807082 | OR786394 | OR786416 | OR826374 | OR807104 |  |
| *Eriosyce odieri* | BV241 | Monte Amargo | OR807083 | OR786395 | OR786417 | OR826375 | OR807105 |  |
| *Eriosyce taltalensis* | MR9488 | Taltal, Cerro Perales | OR807084 | OR786396 | OR786418 | OR826376 | OR807106 |  |
|  |  |  |  |  |  |  |  |  |
